# Prevalence and Risk Factors of Cardiovascular Disease in Chronic Kidney Disease Patients at King Abdulaziz University Hospital (KAUH)

**DOI:** 10.7759/cureus.57409

**Published:** 2024-04-01

**Authors:** Rana A Nabalawi, Mohammed Abdullah Bamuflih, Abdullah Alaa Farid, Khalid Ghali Almramhi, Muhannad Salem Dawood, Mohammad Salah Ahmed, Khaled S Alfawaz, Abdulaziz Mustafa Adnan

**Affiliations:** 1 Nephrology, Faculty of Medicine, King Abdulaziz University, Jeddah, SAU; 2 Faculty of Medicine, King Abdulaziz University, Jeddah, SAU; 3 Internal Medicine, Faculty of Medicine, King Abdulaziz University, Jeddah, SAU; 4 Faculty of Medicine, King Abdulaziz University Hospital, Jeddah, SAU

**Keywords:** cardiovascular medicine, cardio-oncology, hypertensive heart disease, cardiac mri, renal injury, cardiovascular disease and kidney injury, kidney disease and heart disease, kidney failure, heart disease, chronic kidney disease (ckd)

## Abstract

Background: Chronic kidney disease (CKD) has emerged as a significant global health concern, with its incidence doubling over the past three decades. Cardiovascular diseases (CVD) pose a major threat to CKD patients, surpassing the risk of progressing to end-stage renal disease. While previous studies worldwide have shed light on this association, limited research has been conducted in Saudi Arabia regarding this burden. This study aims to fill this gap by identifying the prevalence and risk factors of CVD in CKD patients at King Abdulaziz University Hospital (KAUH), Jeddah, Saudi Arabia, between 2017 and 2022.

Methods: A six-year retrospective review of medical records at KAUH was conducted, including 465 non-end-stage CKD patients aged 30 to 79. Data, including demographics, clinical information, and laboratory results, were collected and statistically analyzed to investigate the association between variables.

Results: Out of 465 CKD patients, 262 (56.3%) were diagnosed with CVD, with congestive heart failure and ischemic heart disease being the most common types. The majority were male 259 (55.7%), non-Saudi 278 (59.8%), and aged 60 years and older 291 (62.6%). Hypertension 394 (84.7%) and diabetes mellitus 336 (72.3%) were prevalent comorbidities. Severely reduced left ventricular ejection fraction, proteinuria, diabetes mellitus, and higher BMI were identified as significant risk factors for CVD in CKD patients.

Conclusion: This research contributes valuable insights into the prevalence and risk factors of CVD in CKD patients in Saudi Arabia, emphasizing the need for early detection and intervention. The identified risk factors provide a basis for developing targeted preventive strategies to mitigate this population's CVD burden.

## Introduction

Chronic kidney disease (CKD) is a global health issue due to its prevalence and incidence almost doubling during the last three decades and it affects 15-20% of adults worldwide [1,2]. CKD is defined as the presence of an estimated glomerular filtration rate (eGFR) < 60 ml/min/1.73 m2 [2]. CKD patients experience an increased risk of diverse adverse outcomes; however, cardiovascular diseases (CVD) are of particular relevance as it is the major cause of morbidity and mortality in these patients [1]. There is a broad spectrum of CVD associated with CKD, including ischemic heart disease, congestive heart failure, arrhythmia, left ventricular hypertrophy, and peripheral artery disease [3]. In this patient population, dying from CVD is exponentially greater than the patient reaching end-stage renal disease [4]. It is a belief that CKD is a cardiovascular disease equivalent [5]. Additionally, there could be risk factors that are common between CKD and CVD, such as Diabetes and Hypertension [1].

Several previous studies elucidated in various nations throughout the world shed light on this topic. One of the studies conducted in 2022 concluded that CKD patients have an increased risk for CVD and that it is the leading cause of death in this population [1]. A study that was done in China in 2017 also showed similar results that a higher chance of CVD development in CKD patients was associated with factors such as age, comorbidities like hypertension and diabetes, and lower eGFR [6]. A study from Sri Lanka done in the year 2016-2017 reported that 39 out of the total 132 CKD patients had coronary artery events [3]. Adding to this, a Malaysian study showed that CKD patients developing CVD were older and that their systolic pressure was higher than non-CVD patients [5].

Unfortunately, no single biomarker has been used as a diagnostic biomarker for CVD in CKD patients in a clinical situation because CKD is a heterogeneous disease with several comorbidities. The identification of CVD in CKD patients typically occurs too late, when symptoms are already present. Therefore, it's crucial to determine reliable CVD biomarkers in CKD patients so that early prevention and therapy may be implemented.

Furthermore, as far as we are aware there is limited research done in the Middle East, specifically in the Kingdom of Saudi Arabia (KSA) studying the relationship of CVD in CKD patients. Thus, the purpose of this study is to identify the prevalence and risk factors of CVD in CKD patients during 2017-2022 at King Abdulaziz University Hospital (KAUH), Jeddah, Saudi Arabia.

## Materials and methods

This six-year retrospective record review was conducted from 2017 to 2022 after receiving approval from the research ethical committee at the College of Medicine, King Abdulaziz University Hospital (KAUH), Jeddah, Kingdom of Saudi Arabia (Reference: 31-23). We included patients diagnosed with non-end-stage CKD with or without proteinuria, aged between 30 and 79 years, from 2017 to 2022 at KAUH. All patients aged less than 30 years and older than 79 years, patients without evidence of CKD, and dialysis patients were excluded. The data was collected manually from our hospital information system, Phoenix, using Google Forms. We extracted the following variables from 465 patients: medical record number, nationality, gender, age, BMI, any associated co-morbidities (diabetes mellitus, hypertension, dyslipidemia, anemia, cerebrovascular accident, thyroid disease, SLE, chronic liver disease, malignancy), smoking status, medications, presence of CVD (IHD, CHF, LVH, valvular disease, left ventricular ejection fraction), lab assessment: hemoglobin, troponin I, creatinine, urea, blood pH, hemoglobinA1C, triglycerides, total cholesterol, LDL cholesterol, HDL cholesterol, and proteinuria. Microsoft Excel v16.0 (Microsoft Corporation, Redmond, USA) was used to organize the data, which was then analyzed statistically using IBM SPSS Statistics for Windows, Version 26 (Released 2019; IBM Corp., Armonk, New York, United States). To investigate the association between the variables, the Chi-squared test (χ2) was applied to categorical variables that were expressed as frequencies and percentages. The association between the numerical parametric variables that were presented as mean and standard deviation (Mean ± SD) was examined using the Mann-Whitney U test. Regression outcomes were expressed as odds ratios (ORs) at confidence intervals (CIs) of 95%. For all tests, a p-value of <0.05 was deemed significant.

## Results

The records of 465 patients were analyzed in this retrospective study. As shown in Table 1, most of our patients were males 259 (55.7%) and non-Saudis 278 (59.8%). The majority of CKD patients included were 60 years and older 291 (62.6%) followed by patients aged between 50 and 59 (96 patients; 20.6%).

**Table 1:** Distribution of patients according to their demographics (N.: 465). Data has been represented as Numbers (N) & Percentages (%).

Variable	N. (%)
Age	
30-49	78 (16.8)
50-59	96 (20.6)
60-69	138 (29.7)
70-79	153 (32.9)
Gender	
Female	206 (44.3)
Male	259 (55.7)
Nationality	
Non-Saudi	278 (59.8)
Saudi	187 (40.2)

Out of the 465 patients, 214 (46%) had proteinuria, 112 (24.1%) of them had a severely elevated level which is more than 50mg/mmol, followed by 55 (11.8%) which are patients who had a moderately elevated level which is between 15 and 50mg/mmol and 48 (10.3%) had a mild increase with a proteinuria level of <15mg/mmol (Table 2). When it comes to left ventricular ejection fraction, as shown in Table 2, 110 (23.7%) had a normal ejection fraction of >55%, 94 (20.2%) had an ejection fraction between 40 and 55%, while 70 (15.1%) had an ejection fraction of less than 30%. Moreover, the majority of our patients were hypertensive 394 (84.7%), while 336 (72.3%) had diabetes mellitus and 65 (14%) experienced a previous cerebrovascular accident, as shown in Table 2.

**Table 2:** Distribution of patients according to their clinical data and chronic diseases (N.: 465). Data has been represented as Numbers (N) & Percentages (%). LVEF: Left ventricular ejection fraction; DM: diabetes mellitus; HTN: hypertension; CVA: cerebrovascular accident; CLD: chronic liver disease; SLE: systemic lupus erythematosus.

Variable	N. (%)
LVEF	
<30% (Severe)	70 (15.1)
30%-40% (Moderate)	60 (12.9)
40%-55% (Mild)	94 (20.2)
>55% (Normal)	110 (23.7)
Proteinuria	
No	251 (53)
Yes	214 (46)
Proteinuria level	
<15 mg/mmol (Mildly elevated)	48 (10.3)
15-50 mg/mmol (Moderately elevated)	55 (11.8)
>50 mg/mmol (Severely elevated)	112 (24.1)
Chronic diseases	
DM	
No	129 (27.2)
Yes	336 (72.3)
HTN	
No	71 (15.3)
Yes	394 (84.7)
CVA	
No	400 (86)
Yes	65 (14)
Dyslipidemia	
No	405 (87.1)
Yes	60 (12.9)
Anemia	
No	433 (93.1)
Yes	32 (6.9)
Thyroid Disorders	
No	420 (90.3)
Yes	42 (9)
On thyroid medications	3 (0.6)
Malignancy	
No	436 (93.8)
Yes	29 (6.2)
Smoking	
No	421 (92.7)
Yes	34 (7.3)
Medications	
No	414 (89)
Yes	51 (11)
CLD	
No	443 (95.3)
Yes	22 (4.7)
SLE	
No	457 (98.3)
Yes	8 (1.7)

**Figure 1:**
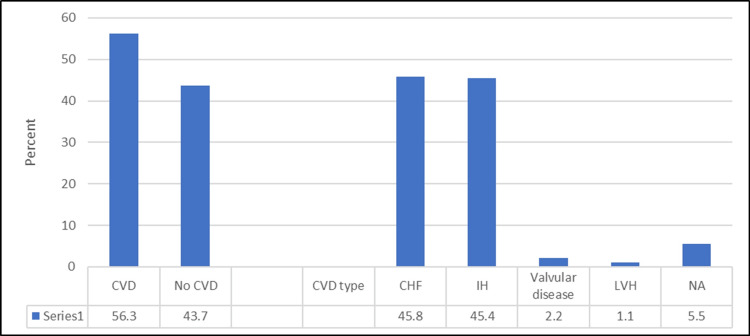
Percentage distribution of the prevalence of CVD (N.: 465) and its types (N.: 262). Data has been represented as Percentages (%). CVD: Cardiovascular disease; CHF: congestive heart failure; IHD: ischemic heart disease; LVH: left ventricular hypertrophy; NA: not applicable.

With regard to heart disease, 262 patients (56.3%) were found to have been diagnosed with it; 120 (45.8%) of them had congestive heart failure, 119 (45.4%) had ischemic heart disease, while 6 (2.2%) were found to have valvular heart disease and 3 (1.1%) had left ventricular hypertrophy, (Figure 1). 

Table 3 outlines the median and interquartile range of the laboratory workup, as the median creatinine level was 282 μmol (IQR:243), while hemoglobin A1C (HbA1C) was 6.9 mmol (IQR:3) and the median troponin levels were 0.08 ng/ml (IQR:0.01).

**Table 3:** Median (IQR) levels of laboratory data (N.:465). Data has been represented as Median, Interquartile range. LDL: Low-density lipoprotein; HDL: high-density lipoprotein

Variable	Median
BMI	27.73 (9.28)
Hemoglobin	8.95 (2.7)
Troponin	0.08 (0.01)
Creatinine	282 (243)
Urea	17.9 (17.4)
PH	7.35 (0.01)
HbA1C	6.9 (3)
Triglycerides	1.22 (1)
Total Cholesterol	3.36 (1)
LDL Cholesterol	2.07 (1)
HDL Cholesterol	0.95 (0.01)

The relationship between CVD prevalence and patients’ demographic data is shown in Table 4. Regarding patients aged between 30 and 49 years, 21 (8%) had CVD; this is considered a significant finding represented by the p-value (<0.001). CVD prevalence in other age groups was found to be 52 (19.8%), 83 (31.7%), and 106 (40.5%) for the age groups 50-59, 60-69, and 70-79, respectively. Results showed that 139 male patients were diagnosed with CVD while only 123 females. Most of the CVD patients were non-Saudi 161 and only 101 were Saudis.

**Table 4:** Relationship between CVD prevalence and patients' demographic data (No: 465). Data has been represented as numbers (N) & percentages (%). A significant p-value is considered (p<0.05). CVD: Cardiovascular disease

Variable	CVD		X^2^	p-value
	With CVD N. (%)	Without CVD N. (%)		
Age			38.85	<0.001
30-49	21 (8)	57 (28.1)		
50-59	52 (19.8)	44 (21.7)		
60-69	83 (31.7)	55 (27.1)		
70-79	106 (40.5)	47 (23.2)		
Gender			1.7	0.192
Female	123 (46.9)	83 (40.9)		
Male	139 (53.1)	120 (59.1)		
Nationality			0.69	0.405
Non-Saudi	161 (61.5)	117 (57.6)		
Saudi	101 (38.5)	86 (42.4)		

Table 5 depicts the relationship between CVD prevalence and patients’ clinical data and chronic diseases. Out of the 465 studied, 66 patients were diagnosed with CVD with a severe decrease in LVEF, also 66 patients had a mild decrease in LVEF and were diagnosed with CVD. In addition to that, a total of 52 and 49 patients with moderate and normal LVEF respectively had been found to have CVD. On the other hand, only 29 patients were diagnosed with CVD with no LVEF being recorded. A sum of 214 patients were found to have proteinuria out of which 133 patients were diagnosed with CVD, as well as that 129 patients were found to have CVD without proteinuria. Regarding proteinuria levels, 112 patients were found to have severely elevated levels out of which 72 patients had CVD. Furthermore, 30 CVD patients had moderately elevated proteinuria levels, and 25 patients were showing such levels as well but without being diagnosed with CVD. A normal to mild increase in proteinuria levels was seen in 31 CVD patients and 17 non-CVD patients. For a total of 250 patients, proteinuria levels were not recorded. As for chronic diseases, 217 CVD patients were found to be diabetic, and a total of 241 CVD-diagnosed patients were hypertensive. CVD patients with a history of CVA and dyslipidemia were 34 and 45 patients respectively. The number of CVD patients with anemia was found to be 16 and having thyroid disorders were 24 patients with only two patients being on thyroid medications. There were 22 smoker patients diagnosed with CVD and 20 CVD patients being on medications at the time of this study. CVD patients having a malignancy were eight patients, and only four CVD patients were diagnosed with CLD. Just one CVD patient was found to have SLE in our study.

**Table 5:** Relationship between CVD prevalence and patients' clinical data and chronic diseases (No: 465). Data has been represented as numbers (N) & percentages (%). A significant p-value is considered (p<0.05). LVEF: Left ventricular ejection fraction; DM: diabetes mellitus; HTN: hypertension; CVA: cerebrovascular accident; CLD: chronic liver disease; SLE: systemic lupus erythematosus; CVD: cardiovascular disease

Variable	CVD		X^2^	p-value
	With CVD N. (%)	Without CVD N. (%)		
LVEF			19.28	<0.001
<30% (Severe)	66 (25.2)	4 (2)		
30%-40% (Moderate)	52 (19.8)	8 (3.9)		
40%-55% (Mild)	66 (25.2)	28 (13.8)		
>55% (Normal)	49 (18.7)	61 (30)		
Proteinuria			5.43	0.02
No	129 (49.2)	122 (60.1)		
Yes	133 (50.8)	81 (39.9)		
Proteinuria level			6.55	0.087
<15 mg/mmol (Mildly elevated)	31 (11.8)	17 (8.4)		
15-50 mg/mmol (Moderately elevated)	30 (11.5)	25 (12.3)		
>50 mg/mmol (Severely elevated)	72 (27.5)	40 (19.7)		
Chronic diseases				
DM			33.42	<0.001
No	45 (17.2)	84 (41.4)		
Yes	217 (82.8)	119 (58.6)		
HTN			24.4	<0.001
No	21 (8)	50 (24.6)		
Yes	241 (92)	153 (75.4)		
CVA			0.5	0.479
No	228 (87)	172 (84.7)		
Yes	34 (13)	31 (15.3)		
Dyslipidemia			9.74	0.002
No	217 (82.8)	188 (92.6)		
Yes	45 (17.2)	15 (7.4)		
Anemia			0.56	0.453
No	246 (93.9)	187 (92.1)		
Yes	16 (6.1)	16 (7.9)		
Thyroid disorders			0.14	0.93
No	236 (90.1)	184 (90.6)		
Yes	24 (9.2)	18 (8.9)		
On thyroid medications	2 (0.8)	1 (0.5)		
Malignancy			10.39	0.001
No	254 (96.9)	182 (89.7)		
Yes	8 (3.1)	21 (10.3)		
Smoking			1.04	0.307
No	240 (91.6)	191 (94.1)		
Yes	22 (8.4)	12 (5.9)		
Medications			6.83	0.009
No	242 (92.4)	172 (84.7)		
Yes	20 (7.6)	31 (15.3)		
CLD			13.67	<0.001
No	258 (98.5)	185 (91.1)		
Yes	4 (1.5)	18 (8.9)		
SLE			6.36	0.012
No	261 (99.6)	196 (96.6)		
Yes	1 (0.4)	7 (3.4)		

Table 6 shows the relationship between CVD prevalence and patients laboratory data. BMI and CVD showed a direct significant relation (p-value 0.007). CVD patients were found to have a significantly higher mean troponin level when compared to non-CVD patients (p-value <0.001). A higher mean in CVD patients was also found in the following laboratory values urea, PH, HbA1C, and HDL cholesterol with PH and HbA1C having significant correlations to CVD with p-values of 0.013 and <0.001, respectively. On the other hand, triglycerides, total cholesterol, and LDL cholesterol all showed higher mean values in non-CVD patients with triglycerides having a p-value of 0.002.

**Table 6:** Relationship between CVD prevalence and patients' laboratory data (N.: 465). Data has been represented as Mean+/- SD; a significant p-value is considered (p<0.05). CVD: Cardiovascular disease; LDL: low-density lipoprotein; HDL: high-density lipoprotein

Variable	CVD		Mann-Whitney test	p-value
	With CVD mean SD	Without CVD mean SD		
BMI	29.5 ± 6.73	28.06 ± 7.75	2.67	0.007
Hemoglobin	9.78 ± 2.44	9.41 ± 2.48	1.32	0.184
Troponin	2.56 ± 13.61	0.53 ± 3.53	4.32	<0.001
Creatinine	327.59 ± 216.7	412.72 ± 335.12	1.52	0.128
Urea	21.8 ± 13.12	21.01 ± 15.77	1.5	0.131
PH	7.33 ± 0.11	7.3 ± 0.12	2.48	0.013
HbA1C	7.61 ± 2.18	6.87 ± 1.931	3.79	<0.001
Triglycerides	1.145 ± 0.88	1.72 ± 1.09	3.02	0.002
Total Cholesterol	3.39 ± 1.32	3.55 ± 1.44	0.86	0.3861
LDL Cholesterol	2.22 ± 1.05	3.39 ± 1.14	1.03	0.299
HDL Cholesterol	0.97 ± 0.35	0.96 ± 0.34	0.05	0.959

The baseline prevalence of proteinuria in CVD is shown in Table 7. The overall prevalence of proteinuria in CVD was 121 (48.2%) while the percentages in congestive heart failure (CHF), ischemic heart disease (IHD), left ventricular hypertrophy (LVH), and valvular disease were 61 (24.3%), 52 (20.7%), 2 (0.8%), and 6 (2.4%) respectively. The prevalence of proteinuria in CHF was significantly higher than that of other cardiovascular events. The proportion of proteinuria in CHF and IHD was significantly higher than in LVH and valvular disease (p-value 0.016).

**Table 7:** Relationship between the prevalence of proteinuria and types of CVD. Data has been represented as numbers (N) & percentages (%); a significant p-value is considered (p<0.05). CHF: Congestive heart failure; IHD: ischemic heart disease; LVH: left ventricular hypertrophy; CVD: cardiovascular disease

Variable	Proteinuria		χ2	p-value
	CVD with Proteinuria N. (%)	CVD without Proteinuria N. (%)		
CHF	61 (24.3)	59 (27.6)	13.96	0.016
IHD	52 (20.7)	67 (31.3)		
LVH	2 (0.8)	1 (0.5)		
Valvular diseases	6 (2.4)	0 (0.0)		

The results of multiple logistic regression analysis of the risk factors for CVD prevalence are shown in Table 8. The ORs were adjusted at a CI of 95% to assess all potential risk factors listed in the table. In multivariable analysis, the variables significantly associated with the presence of CVD were severe LVEF, having proteinuria especially severely elevated proteinuria, diabetes mellitus, and higher mean BMI. The risk factors of CVD with higher ORs were severe LVEF (OR: 0.64; 95% CI: 0.42-0.67) (p=0.039), followed by having proteinuria (OR: 0.04; 95% CI: 0.003-0.81) (p=0.036), (OR: 2.85; 95% CI: 1.01-8.04) in severely elevated proteinuria (p=0.046), diabetes mellitus (OR: 0.12; 95% CI: 0.02-0.52) (p=0.005), and high BMI (OR: 0.85; 95% CI: 0.76-0.96) (p=0.009).

**Table 8:** Multivariate logistic regression analysis of the risk factors (independent predictors) of CVD among studied patients (N.:465). Data has been represented as odds ratio; a significant p-value is considered (p<0.05). LVEF: Left ventricular ejection fraction; DM: diabetes mellitus; HTN: hypertension; CVA: cerebrovascular accident; CLD: chronic liver disease; SLE: systemic lupus erythematosus; LDL: low-density lipoprotein; HDL: high-density lipoprotein

Variable	B	Wald	p-value	Odds Ratio (CI:95%)
Age	0.01	0.002	0.967	1.01 (0.57-1.77)
Gender	0.47	0.54	0.461	1.6 (0.45-5.64)
Nationality	0.32	0.27	0.599	0.72 (0.21-2.43)
LVEF	0.44	4.28	0.039	0.64 (0.42-0.67)
Proteinuria	3.01	4.42	0.036	0.04 (0.003-0.81)
Proteinuria level	1.05	3.96	0.046	2.85 (1.01-8.04)
DM	2.08	7.98	0.005	0.12 (0.02-0.52)
HTN	1.51	1.77	0.183	0.21 (0.02-2.05)
CVA	0.58	0.55	0.456	1.78 (0.38-8.24)
Dyslipidemia	0.62	0.43	0.511	1.86 (0.29-12.03)
Anemia	1.45	0.96	0.326	0.23 (0.01-4.25)
Thyroid disorders	0.68	0.49	0.48	0.5 (0.07-3.38)
Malignancy	0.72	0.33	0.565	2.05 (0.17-24.13)
Smoking	0.49	0.14	0.704	0.61 (0.04-7.63)
Medications	0.85	1.16	0.465	0.42 (0.4-4.2)
CLD	0.13	1.4	0.542	0.8 (0.1-2.13)
SLE	0.04	0.03	0.781	0.9 (0.1301.87)
BMI	0.15	6.82	0.009	0.85 (0.76-96)
Hemoglobin	0.07	0.16	0.688	0.92 (0.64-1.33)
Troponin	0.5	1.64	0.2	0.6 (0.28-1.3)
Creatinine	0.001	0.004	0.947	0.1 (0.99-1)
Urea	0.07	3.66	0.055	0.92 (0.85-1)
PH	0.1	0.001	0.976	0.03 (0.6-1.2)
HbA1C	0.05	0.07	0.788	0.94 (0.64-1.39)
Triglycerides	0.3	1.01	0.314	1.63 (0.74-2.48)
Total Cholesterol	0.47	0.77	0.37	1.6 (0.55-4.6)
LDL Cholesterol	0.62	0.96	0.326	0.53 (0.15-1.85)
HDL Cholesterol	1.24	0.95	0.328	0.48 (0.28-2.37)

## Discussion

One of the most important findings in our study was that the prevalence of cardiovascular disease in CKD patients was 56.1%. This is higher than that in a study performed in China, where CVD prevalence among CKD patients was found to be 9.8%, this finding could be due to the older mean age of the studied population in our study. With congestive heart failure and ischemic heart disease being the most common 45.8% and 45.4%, respectively, in our data in comparison to 9% for HF and 20.58% for ischemic heart disease [6]. The majority of our population was male (55.7%) and had an elevated BMI of 28.87 ± 7.22 kg/m2, which was in parallel with a study done in China in the period of 2011 to 2016 with a result of 59.22 and 24.47 ± 3.63, respectively. On the other hand, our patients had a higher age range than the Jun Yuan study [6]. In terms of risk factors, the majority of our patients were hypertensive (84.7%), while 336 (72.3%) had diabetes mellitus. This correlates with a research study done by our colleagues in 2022 that showed the most common comorbidity to be HTN 409 (72.5%), followed by DM 392 (69.5%) patients [7].

An important result of our study showed that the prevalence of CVD in CKD patients was higher among patients aged between 30-49 years. However, when relating this to another study that was done in Germany, it showed that the prevalence of CVD in CKD patients was in an older age group. Moreover, our results showed that prevalence is higher in male patients who were diagnosed with CVD. This matches a German study, that revealed CKD male patients were more involved in the prevalence of CVD [8].

The most frequent cardiac issue in patients with CKD was reduced LVEF, which is associated with a significant risk of cardiovascular death [9]. On the determinants of LVEF in CKD patients in our study, we divided them into degrees based on the LVEF severity. These were normal, mild, moderate, and severe. Out of 456 patients, mild and severe LVEF were the most prevalent in CKD patients, followed by moderate and normal LVEF. In comparison, another study conducted in Sri Lanka showed a decreased prevalence of reduced LVEF in CKD patients [3].

Patients were divided into multiple groups according to the degree of proteinuria. Out of 112 patients, 72 of them showed severely elevated levels and CVD, whereas 30 and 31 showed moderate and mild levels of proteinuria with CVD, respectively. On the other hand, a study was done in Sri Lanka and concluded that proteinuria does not seem to play a significant role in IHD pathogenesis about CKD, this could be due to our study having a more comprehensive approach, and not focusing on IHD as a specific entity [3]. Another study showed similar findings to our research, showing that proteinuria was found in those with a previous history of CVD and were known to have CKD [10].

Apart from a diagnosis of CVD, our participants were found to have a lot of chronic diseases including diabetes, hypertension, CVA, dyslipidemia, anemia, thyroid disorders, CLD, and smoking. These findings correlate to similar results that were found in a Canadian study, as well as a Chinese study [6,8].

Our research found a significant connection between CVD and BMI with a p-value of 0.007. This link between CVD and BMI agrees with previous results from a study that was conducted in the USA and showed that a higher BMI increases the future risk of CVD development [11].

Furthermore, HBA1C was markedly noticed to have an association with CVD, P-value (<0.001). A previous research study done in Japan in 2015, stated that elevated levels of HbA1c increase the chance of developing CVD, and this echoes our results [12].

Also, triglyceride levels in non-CVD patients were significantly higher than those of CVD patients with a p-value of (0.002). This result agreed with a previous research study performed in Malaysia in 2018 and showed that triglycerides were elevated in non-CVD patients [5]. This could be explained as individuals with CVD have more control over triglycerides levels than non-CVD patients as they are on medications.

Our study found that severe LVEF was significantly associated with the presence of CVD, and this was observed in 15.1% of the population (p=0.039). This association between reduced LVEF and CVD in CKD patients aligns with previous research studies. For instance, a study by Smith et al. (2013) also reported a significant correlation between reduced LVEF and CVD in CKD patients, highlighting the importance of cardiac evaluation in this population [13].

In addition, our study identified proteinuria as another significant risk factor for CVD in CKD patients. We observed that proteinuria was present in 46% of CKD patients, and severely elevated proteinuria was noted in 24.1% of the population (p=0.036). These findings concur with previous research emphasizing the link between proteinuria and increased cardiovascular risk in CKD patients. Specifically, a study by Matsushita et al. (2015) demonstrated that the presence and severity of proteinuria were independently associated with a higher risk of cardiovascular events among CKD patients [14].

The detection of severe LVEF and proteinuria as significant risk factors for CVD in CKD patients underscores the necessity of comprehensive cardiac and renal evaluations. Early identification and management of these risk factors could potentially mitigate the development and progression of cardiovascular complications.

The main limitation of our study is that it is retrospective in nature and was conducted at a single organization. This made the findings of the study prone to bias and it also limited its generalizability.

## Conclusions

In conclusion, this six-year retrospective study conducted at King Abdulaziz University Hospital (KAUH) in Jeddah, Saudi Arabia, aimed to investigate the prevalence and risk factors of CVD in CKD patients. The research found that CKD patients at KAUH had a high prevalence of CVD, with 56.1% of the studied population affected. This finding is consistent with global trends, highlighting the significant burden of cardiovascular complications in CKD patients. These findings emphasize the importance of managing these modifiable risk factors in CKD patients to mitigate the progression of CVD.
